# The mGlu5 receptor negative allosteric modulator mavoglurant reduces escalated cocaine self-administration in male and female rats

**DOI:** 10.1007/s00213-024-06634-5

**Published:** 2024-06-13

**Authors:** Leandro F. Vendruscolo, Janaina C.M. Vendruscolo, Kimberly E. Whiting, Jane B. Acri, Nora D. Volkow, George F. Koob

**Affiliations:** 1 Stress and Addiction Neuroscience Unit, Integrative Neuroscience Research Branch, National Institute on Drug Abuse, Intramural Research Program, National Institute on Alcohol Abuse and Alcoholism, Division of Intramural Clinical and Biological Research, National Institutes of Health, BRC Room 08A727, 251 Bayview Blvd, Baltimore, MD 21224 USA; 2 Neurobiology of Addiction Section, Integrative Neuroscience Research Branch, National Institute on Drug Abuse, Intramural Research Program, National Institutes of Health, Baltimore, MD 21224 USA; 3 Division of Therapeutics and Medical Consequences, National Institute on Drug Abuse, National Institutes of Health, Bethesda, MD 20892 USA; 4 Laboratory of Neuroimaging, National Institute on Alcohol Abuse and Alcoholism, National Institutes of Health, Bethesda, MD 20892 USA

**Keywords:** Cocaine addiction, Drug addiction, Cocaine dependence, Rodent models

## Abstract

**Rationale:**

Cocaine use disorder (CUD) is a brain disorder for which there is no Food and Drug Administration-approved pharmacological treatment. Evidence suggests that glutamate and metabotropic glutamate receptor subtype 5 (mGlu5) play critical roles in synaptic plasticity, neuronal development, and psychiatric disorders.

**Objective:**

In the present study, we tested the hypothesis that the mGlu5 receptor is functionally involved in intravenous cocaine self-administration and assessed the effects of sex and cocaine exposure history.

**Methods:**

We used a preclinical model of CUD in rats that were allowed long access (LgA; 6 h/day) or short access (ShA; 1 h/day) to intravenous cocaine (750 µg/kg/infusion [0.1 ml]) self-administration. Rats received acute intraperitoneal or oral administration of the mGlu5 receptor negative allosteric modulator mavoglurant (1, 3, and 10 mg/kg) or vehicle.

**Results:**

Both intraperitoneal and oral mavoglurant administration dose-dependently reduced intravenous cocaine self-administration in the first hour and in the entire 6 h session in rats in the LgA group, with no effect on locomotion. In the ShA group, mavoglurant decreased locomotion but had no effects on cocaine self-administration. We did not observe significant sex × treatment interactions.

**Conclusions:**

These findings suggest that the mGlu5 receptor is involved in escalated cocaine self-administration. These findings support the development of clinical trials of mavoglurant to evaluate its potential therapeutic benefits for CUD.

## Introduction

Cocaine use disorder (CUD) is a chronic relapsing disorder with significant deleterious health and societal consequences. Among people 12 years of age and older in 2021, 4.8 million (1.7%) used cocaine in the past year, and 1.8 million used cocaine in the past month. A total of 1.4 million people 12 and older (0.5%) could be classified with CUD in the past year, 40% of whom had severe CUD (Substance Abuse and Mental Health Services Administration, 2022). No pharmacotherapies have yet been approved by the U.S. Food and Drug Administration for the treatment of CUD.

The excitatory neurotransmitter glutamate and metabotropic glutamate receptor subtype 5 (mGlu5 receptor) have been hypothesized to play critical roles in drug seeking that is associated with cocaine addiction (Pomierny-Chamiolo et al. 2015). Cocaine intoxication causes the release of glutamate in the basal forebrain (Reid et al. 1997); thus, the downregulation of mGlu5 receptor levels may represent a partial adaptation to an increase in glutamate signaling. CUD in humans and cocaine self-administration in rodents were associated with a reduction of mGlu5 receptor levels in the striatum, amygdala, and other cortical and subcortical regions (de Laat et al. 2018; Martinez et al. 2014; Milella et al. 2014; Ben-Shahar et al., 2008; Caffino et al., 2022; Hao et al. 2010; Ghasemzadeh et al., 2011; but see Hulka et al. 2014). Numerous genetic and behavioral pharmacology studies indicated a role for the mGlu5 receptor in cocaine reward, behavioral sensitization, the context-, cue, stress-, and cocaine-induced reinstatement of cocaine seeking, and cocaine self-administration (for review, see Niedzielska-Andres et al. 2021; Schwendt and Knackstedt 2021).

mGlu5 receptor negative allosteric modulators, such as 3-([2-methyl-1,3-thiazol-4-yl]ethynyl)pyridine (MTEP) and 2-methyl-6-(phenylethynyl)pyridine (MPEP), reduced short access (ShA) and extended, long access (LgA) cocaine self-administration (Gould et al. 2016; Hao et al. 2010; Kenny et al. 2005; Tessari et al. 2004). LgA to cocaine has evolved as a valid model of the neuroadaptations that drive the excessive drug consumption that is associated with addiction (Edwards and Koob 2013). Compared with rats that are allowed ShA (1 h/day) to intravenous cocaine self-administration, rats that are allowed LgA (6 h/day) to cocaine exhibit several addiction-like behaviors (George et al. 2014; Guglielmo et al. 2023), such as escalated cocaine intake (Ahmed and Koob 1998), greater motivation for cocaine (Ben-Shahar et al. 2008), greater cue- and cocaine-induced reinstatement (Kippin et al. 2006; Mantsch et al. 2004), and persistent cocaine self-administration despite punishment (Vanderschuren and Everitt 2004).

In the present study, we tested the hypothesis that mavoglurant, which is a selective, brain penetrant, negative mGlu5 receptor allosteric modulator with an improved pharmacokinetic profile (e.g., half-life of ~ 2.9 h) in rats compared with MTEP (Vranesic et al. 2014) would also block LgA intravenous cocaine self-administration. Mavoglurant has been tested in humans for several different conditions, including neuropsychiatric disorders, such as Fragile X syndrome (Hagerman et al. 2018) and levodopa-induced dyskinesia in patients with Parkinson’s disease (Negida et al. 2021). Mavoglurant has also been tested in humans with CUD. It was superior to placebo in the proportion of cocaine use days and positive urine measurements of cocaine’s metabolite benzoylecgonine (ClinicalTrials.gov: NCT03242928).

Because previous studies were mostly conducted in male subjects only, we tested both male and female rats. Some studies have reported that female rats self-administer more cocaine than male rats (Becker and Koob 2016; Edwards and Koob 2013; Towers et al. 2023). To our knowledge, the effect of mavoglurant on cocaine self-administration has not been previously tested in male or female laboratory animals. We hypothesized that mavoglurant would decrease intravenous cocaine self-administration in both male and female rats and that this effect would be modulated by a history of extended-access cocaine exposure. The results showed that both intraperitoneal and oral mavoglurant administration dose-dependently reduced intravenous cocaine self-administration in the first hour and in the entire 6 h session in rats in the LgA group, with no effect on locomotion and similar effects in both males and females. In contrast to earlier work with other mGlu5 antagonists, mavoglurant decreased locomotion but had no effects on cocaine self-administration in rats in the ShA group.

## Materials and methods

### Animals

Adult male (*n* = 29) and female (*n* = 25) Wistar rats (Charles River, Raleigh, NC, USA), weighing 175–275 g at the beginning of the experiments, were group-housed (2–4 rats/cage) in a temperature-controlled (22 °C) vivarium on a 12 h/12 h light/dark cycle (lights off at 8:00 AM) with *ad libitum* access to food and water, except during cocaine self-administration sessions. Intravenous cocaine self-administration sessions occurred once per day, 5 days per week, during the dark cycle. The rats were acclimated to the animal facility for at least 7 days before surgery. All procedures adhered to the National Institutes of Health Guide for the Care and Use of Laboratory Animals and were approved by the Institutional Animal Care and Use Committee of the National Institute on Drug Abuse (NIDA) Intramural Research Program.

### Intravenous catheter implantation surgery

The rats were anesthetized with isoflurane (1.5–2.5%) and prepared with chronic indwelling jugular vein catheters as previously described (Carmack et al. 2022). The catheters were flushed daily with heparinized sterile saline (0.2 ml; 30 USP units/ml). The rats were allowed 5–7 days to recover from surgery before behavioral testing.

### Intravenous cocaine self-administration

Cocaine hydrochloride was synthesized and provided by RTI International (Research Triangle Park, NC, USA), NIDA drug supply program. The NIDA Intramural Research Program pharmacy dispensed cocaine that was dissolved in physiological saline. Intravenous cocaine self-administration sessions were conducted in standard operant conditioning chambers (30.5 cm × 24.1 cm × 21.0 cm; Med Associates, St. Albans, VT, USA). The operant chambers were housed inside light- and sound-attenuating wood chambers. The rats were trained to self-administer cocaine (750 $\mu$g/kg/infusion, 0.1 ml) in 1 h sessions under a fixed-ratio 1 (FR1) schedule of reinforcement, in which each active lever press resulted in cocaine delivery over 2.3 s. A stimulus light above the active lever turned on for a 20-s timeout period at the onset of each infusion, during which time responses on the active lever had no programmed consequences. Lever presses on the inactive lever were recorded but had no programmed consequences. After 12 to 15 acquisition sessions of cocaine self-administration, the rats were split into ShA (1 h) and LgA (6 h) groups. Rats in the LgA groups underwent 12 (6 h) escalation sessions of FR1 cocaine self-administration, and rats in the ShA group continued with 1 h (FR1) self-administration sessions before mavoglurant testing.

### Mavoglurant testing

For intraperitoneal administration, mavoglurant was synthesized and provided by RTI International (Research Triangle Park, NC, USA), NIDA drug supply program, and dissolved in 10% Kolliphor and 10% dimethylsulfoxide, diluted with saline, and administered at doses of 0, 1, 3, 10 mg/kg 30 min before behavioral testing. For oral administration, mavoglurant was purchased from MedChemExpress (catalog no. HY-15,257, Monmouth Junction, NJ, USA) and suspended in 0.5% methylcellulose in water and administered at doses of 0, 1, 3, 10 mg/kg 60 min before behavioral testing. The mavoglurant doses and pretreatment time were based on previous pharmacokinetic studies that used intravenous and oral routes of administration (Vranesic et al. 2014). The rats were habituated to gavage or intraperitoneal injections of vehicle before drug testing. For cocaine self-administration testing, the rats received all doses in a within-subjects Latin-square design. A cocaine self-administration session without mavoglurant treatment was performed between testing days. During mavoglurant testing, locomotion was measured in the operant chambers by four infrared beams that were evenly spaced horizontally (6 cm apart) and 3 cm above the grid floor. Locomotion (crossovers) was counted when a rat crossed from one side of the chamber to the opposite side (i.e., crossing all beams).

### Statistical analysis

We identified outliers using Grubb’s test with α = 0.05 applied to each variable separately and treated outliers as previously published (Marchette et al. 2023). Briefly, for cocaine self-administration prior to mavoglurant testing, excluded outliers were replaced by the average of temporally proximal values for each animal (16 of 996 data points; 1.6%). During mavoglurant testing, datasets from one male and one female rat in the LgA group for locomotion were excluded from the analysis (i.e., *≥* 25% of the data were identified as outliers). The data are shown without outliers.

For the cocaine self-administration data before mavoglurant testing, we analyzed the ShA and LgA group data separately using two-way repeated-measures analysis of variance (ANOVA), with sex as the between-subjects factor and session as the within-subjects factor. For effects of mavoglurant, we analyzed ShA and LgA group data separately using two-way repeated-measures ANOVA, with sex as the between-subjects factor and treatment as the within-subjects factor. We also conducted a three-way ANOVA, with Sex as the between-subjects factor and Treatment and Time as within-subjects factors, to analyze the time-course of effects of intraperitoneal mavoglurant administration in the LgA group. We conducted *post hoc* comparisons using Duncan’s test when a main effect of a factor with more than two levels was significant (i.e., Session, Treatment, or Time) and when interactions were significant. The data are expressed as the mean and standard error of the mean (SEM), and individual data points are shown where appropriate. Values of *p* < 0.05 were considered statistically significant for all tests. The analyses were performed using Statistica 13 software (TIBCO Software, Palo Alto, CA, USA) and GraphPad Prism 10.2.1 software (San Diego, CA).

## Results

### ShA - intraperitoneal

**Fig. 1:**
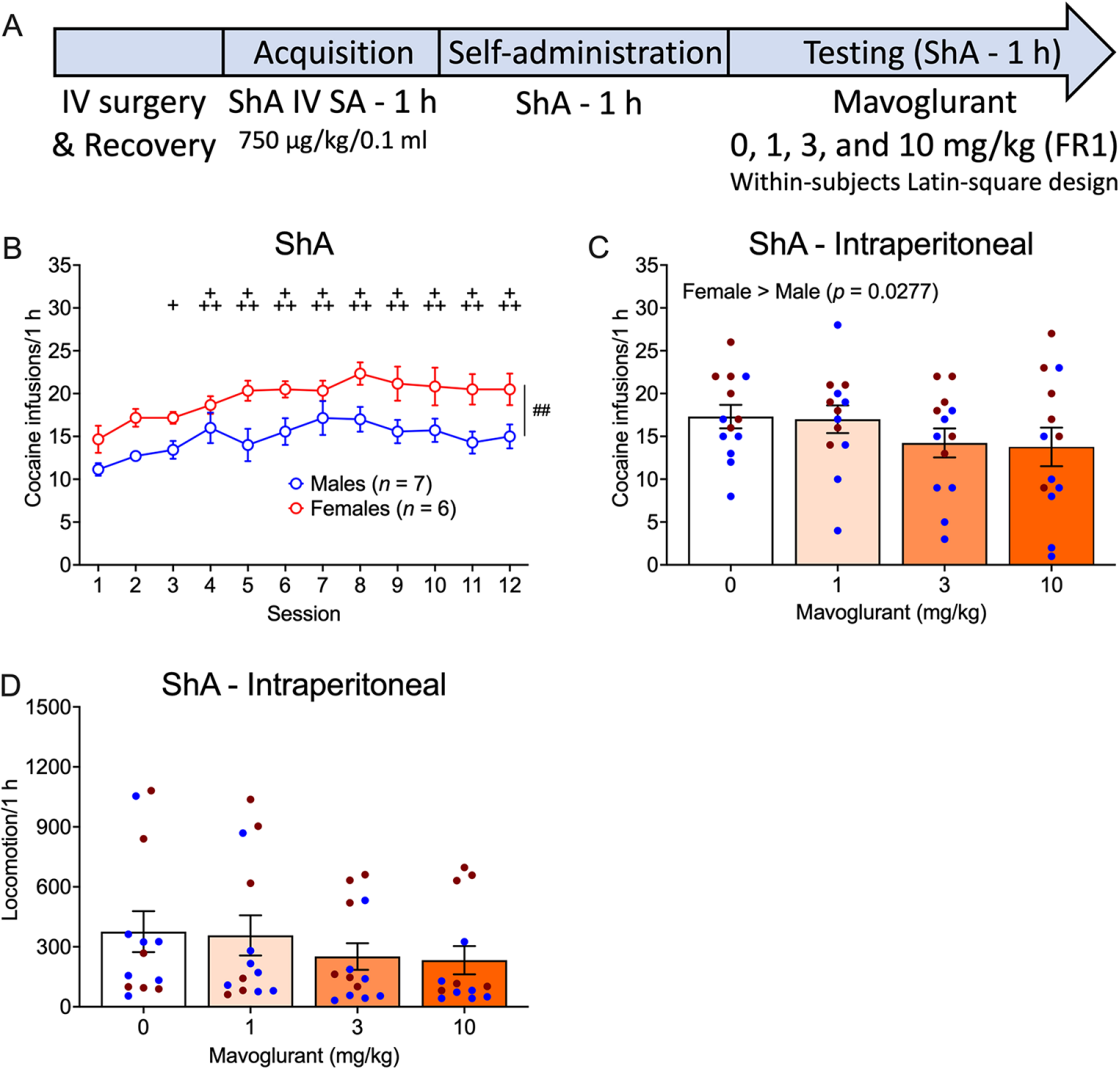
Effect of intraperitoneal mavoglurant administration on intravenous cocaine self-administration under ShA conditions. **(A)** Timeline of the experiment. Acquisition data are not shown. **(B)** Number of cocaine infusions (750 µg/kg/0.1 ml) before mavoglurant testing in male and female rats in the ShA group. **(C)** Number of cocaine infusions in male and female rats following intraperitoneal mavoglurant administration. **(D)** Locomotion in male and female rats following intraperitoneal mavoglurant administration. The data are expressed as the mean ± SEM. *n* = 7 males (blue symbols), 6 females (red symbols); ^+^*p* < 0.05, ^+++^*p* < 0.001, difference from session 1, regardless of sex; ^##^*p* < 0.01, difference between males and females. IV, intravenous; SA, self-administration; ShA, short-access; FR1, fixed-ratio 1

The timeline of the experiment with intraperitoneal mavoglurant administration in the ShA group is shown in Fig. 1A. Female rats self-administered more cocaine than male rats (Sex effect: *F*_1,11_ = 9.66, *p* = 0.0100; η^2^ = 0.47). Cocaine self-administration varied across sessions, regardless of sex (Session effect: _11,121_ = 6.79, *p* < 0.0001; η^2^ = 0.38; Sex x Session interaction: *F*_11,121_ = 0.69, *p* = 0.7503; η^2^ = 0.06). Compared with session 1, the rats self-administered more cocaine (Fig. 1B) in sessions 3 (*p* = 0.0259) and 4–12 (*p* < 0.0001).

During intraperitoneal mavoglurant testing, females self-administered more cocaine than males (Sex effect: *F*_1,11_ = 6.43, *p* = 0.0277; η^2^ = 0.37), but mavoglurant had no effect on cocaine self-administration (Treatment effect: *F*_3,33_ = 2.23, *p* = 0.1028; η^2^ = 0.17; Sex x Treatment interaction: *F*_3,33_ = 1.47, *p* = 0.2410; η^2^ = 0.12; Fig. 1C).

Male and female animals did not differ in locomotion and mavoglurant did not alter locomotion (Sex effect: *F*_1,11_ = 1.63, *p* = 0.2285; η^2^ = 0.13; Treatment effect: *F*_3,33_ = 2.64, *p* = 0.0668; η^2^ = 0.19; Sex x Treatment interaction: *F*_3,30_ = 1.07, *p* = 0.3762; η^2^ = 0.09; Fig. 1D).

### LgA - intraperitoneal

**Fig. 2:**
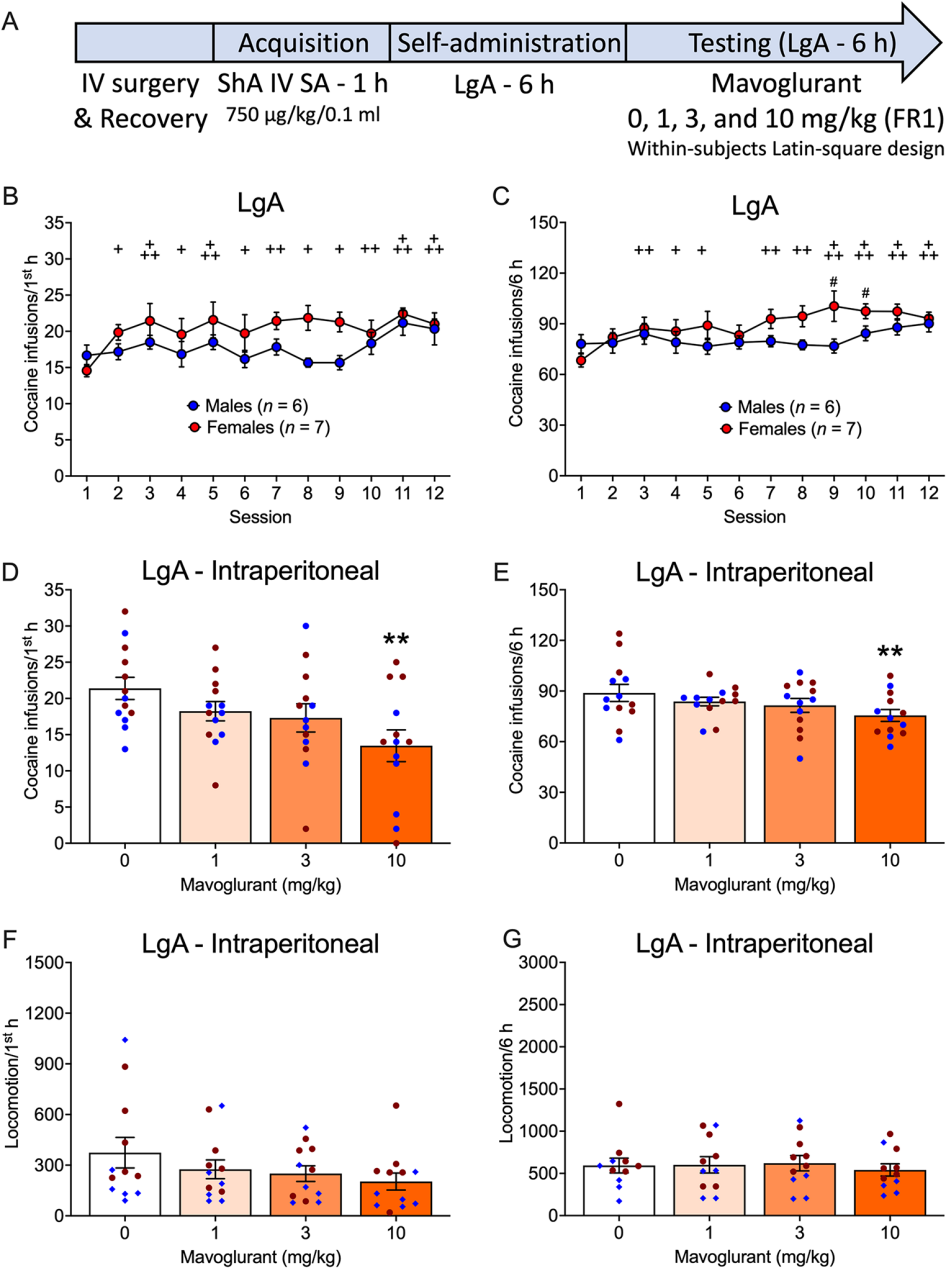
Effect of intraperitoneal mavoglurant administration on intravenous cocaine self-administration under LgA conditions. **A.** Timeline of the experiment. Acquisition data are not shown. **B, C.** Number of cocaine infusions (750 µg/kg/0.1 ml) before mavoglurant testing in male and female rats in the LgA group in the (**B**) first hour and (**C**) entire 6 h sessions. **D, E.** Number of cocaine infusions in the (**D**) first hour and (**E**) entire 6 h sessions in male and female rats following intraperitoneal mavoglurant administration. **F, G.** Locomotion in the (**F)** first hour and (**G**) 6 h in male and female rats following intraperitoneal mavoglurant administration. The data are expressed as the mean ± SEM. *n* = 6 males (blue symbols), 7 females (red symbols). ^+^*p* < 0.05, ^++^*p* < 0.01, ^+++^*p* < 0.001, difference from session 1, regardless of sex; **p < 0.01, difference from 0 mg/kg. IV, intravenous; SA, self-administration; ShA, short-access; FR1, fixed-ratio 1

The timeline of the experiment with intraperitoneal mavoglurant administration in the LgA group is shown in Fig. 2A. Cocaine self-administration varied during the first hour of cocaine self-administration across sessions, but males and females did not differ in cocaine self-administration (Sex effect: *F*_1,11_ = 2.93, *p* = 0.1151; η^2^ = 0.21; Session effect: *F*_11,121_ = 3.27, *p* < 0.0006; η^2^ = 0.23; Sex x Session interaction: *F*_11,121_ = 1.60, *p* = 0.1070; η^2^ = 0.13; Fig. 2B). Compared with session 1, rats self-administered more cocaine in all sessions (*p* < 0.05-0.0001).

During the entire 6 h sessions, males and females differed in cocaine self-administration and cocaine varied across sessions (Sex effect: *F*_1,11_ = 1.81, *p* = 0.2051; η^2^ = 0.14; Session effect: *F*_11,121_ = 4.15, *p* < 0.0001; η^2^ = 0.27; Sex x Session interaction: *F*_11,121_ = 2.45, *p* = 0.0086; η^2^ = 0.18; Fig. 2C). Compared with session 1, rats self-administered more cocaine in sessions 3 (*p* = 0.0063), 4 (*p* = 0.0385), 5 (*p* = 0.0284), and 7–12 (*p* < 0.01-0.0001). Females self-administered more cocaine than males in sessions 9 (*p* = 0.0369) and 10 (*p* = 0.0313).

Intraperitoneal mavoglurant administration decreased cocaine self-administration in the first hour of cocaine self-administration, but males and females did not differ (Sex effect: *F*_1,11_ = 1.61, *p* = 0.2304; η^2^ = 0.13; Treatment effect: *F*_3,33_ = 4.81, *p* = 0.0069; η^2^ = 0.30; Sex x Treatment interaction: *F*_3,33_ = 1.08, *p* = 0.3719; η^2^ = 0.09). Intraperitoneal mavoglurant at 10 mg/kg (*p* = 0.0013) decreased cocaine self-administration compared with 0 mg/kg in the first hour (Fig. 2D).

Similar results were found for the entire 6 h sessions (Sex effect: *F*_1,11_ = 0.49, *p* = 0.4995; η^2^ = 0.04; Treatment effect: *F*_3,33_ = 4.00, *p* = 0.0156; η^2^ = 0.27; Sex x Treatment interaction: *F*_3,33_ = 0.32, *p* = 0.8106; η^2^ = 0.04). Mavoglurant at 10 mg/kg reduced cocaine self-administration (*p* = 0.0028) compared with 0 mg/kg (Fig. 2E).

Mavoglurant tended to reduce locomotion in rats in the LgA group in the first hour of cocaine self-administration (Sex effect: *F*_1,10_ = 1.33, *p* = 0.2750; η^2^ = 0.12; Treatment effect: *F*_3,30_ = 2.90, *p* = 0.0510; η^2^ = 0.23; Sex x Treatment interaction: *F*_3,30_ = 0.35, *p* = 0.7863; η^2^ = 0.03; Fig. 2F).

In the entire 6 h sessions, mavoglurant did not affect locomotion (Sex effect: *F*_1,9_ = 2.90, *p* = 0.1216; η^2^ = 0.12; Treatment effect: *F*_3,27_ = 0.33, *p* = 0.8025; η^2^ = 0.04; Sex x Treatment interaction: *F*_3,27_ = 0.20, *p* = 0.8932; η^2^ = 0.02; Fig. 2G).

To investigate temporal effects of mavoglurant, we analyzed the number of cocaine infusions per hour of the 6 h sessions between vehicle and 10 mg/kg mavoglurant (Table 1). We confirmed that mavoglurant reduced cocaine self-administration (Treatment effect: *F*_1,11_ = 7.66, *p* = 0.0183, η^2^ = 0.41). The effect of mavoglurant was more salient in the first hour of the 6 h session (Time x Treatment interaction: *F*_5,55_ = 4.09, *p* = 0.0032, η^2^ = 0.27). There was no Treatment x Sex interaction (*F*_1,11_ = 0.11, *p* = 0.7457, η^2^ = 0.01). Females self-administered more cocaine than males in the first hour (Sex x Time interaction: *F*_5,55_ = 2.76, *p* = 0.0268, η^2^ = 0.20) but not in the entire 6 h session (Sex effect: *F*_1,11_ = 0.94, *p* = 0.3533, η^2^ = 0.08). Cocaine self-administration decreased over time (Time effect: *F*_5,55_ = 9.49, *p* < 0.0001, η^2^ = 0.46). The Treatment x Time x Sex interaction was not significant (*F*_5,55_ = 0.82, *p* = 0.07, η^2^ = 0.49).

**Table 1:** Effect of 10 mg/kg mavoglurant vs. vehicle on intravenous cocaine self-administration over time under LgA conditions

		Time					
Treatment	Sex	1st hour	2nd hour	3rd hour	4th hour	5th hour	6th hour
Vehicle	Male	18.8 ± 2.2	13.7 ± 1.0	12.8 ± 0.8	12.8 ± 0.9	12.7 ± 0.8	13.0 ± 0.4
Vehicle	Female	22.1 ± 2.2	15.0 ± 0.0	14.7 ± 1.4	13.9 ± 1.0	14.1 ± 1.4	12.7 ± 1.1
Mavoglurant	Male	10.2 ± 2.5	13.2 ± 1.2	14.5 ± 2.3	11.0 ± 1.9	12.0 ± 1.0	11.7 ± 1.0
Mavoglurant	Female	13.3 ± 3.2	13.4 ± 0.8	12.3 ± 0.7	12.6 ± 0.5	12.3 ± 0.7	11.3 ± 0.8

The data are expressed as the mean ± SEM. *n* = 6 males, 7 females. Mavoglurant-treated rats self-administered less cocaine compared with vehicle-treated mice in the first hour (*p* < 0.0001) and entire 6 h session (*p* < 0.05). Females self-administered more cocaine compared with males in the first hour (*p* < 0.01)

### ShA - oral

**Fig. 3:**
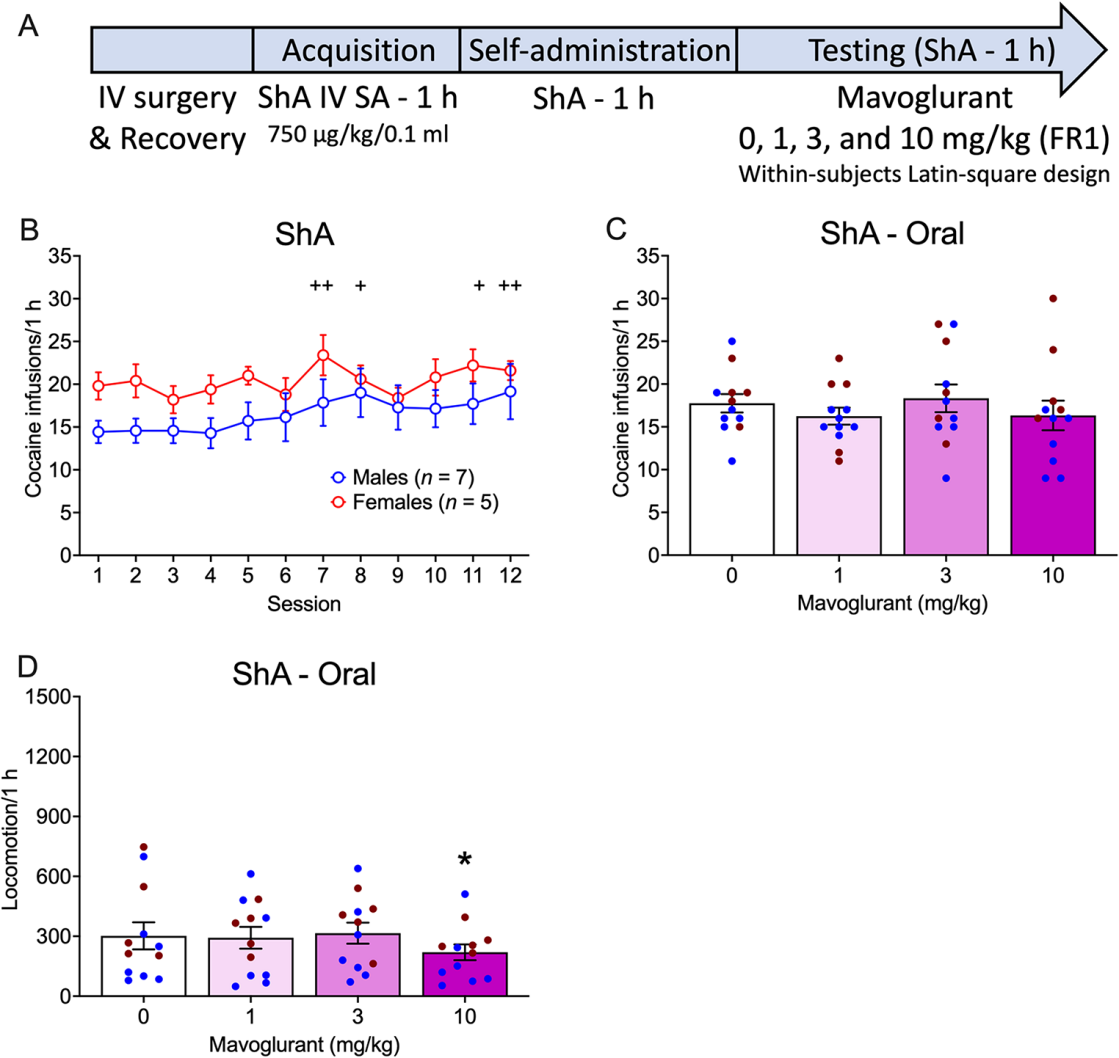
Effect of oral mavoglurant administration on intravenous cocaine self-administration under ShA conditions in rats. **(A)** Timeline of the experiment. Acquisition data are not shown. **(B)** Number of cocaine infusions (750 µg/kg/0.1 ml) before mavoglurant testing in male and female rats in the ShA group. **(C)** Number of cocaine infusions in male and female rats following oral mavoglurant administration. **(D)** Locomotion in male and female rats following oral mavoglurant administration. The data are expressed as the mean ± SEM. *n* = 7 males (blue symbols), 5 females (red symbols). ^+^*p* < 0.05, ^++^*p* < 0.01, difference from session 1, regardless of sex. *p < 0.05, difference from 0 mg/kg. IV, Intravenous; SA, self-administration; ShA, short-access; FR1, fixed-ratio 1

The timeline of the experiment with oral mavoglurant administration in the ShA group is shown in Fig. 3A. Cocaine self-administration varied across sessions, regardless of sex (Sex effect: *F*_1,10_ = 2.00, *p* = 0.1877; η^2^ = 0.17; Session effect: _11,110_ = 3.59, *p* < 0.0002; η^2^ = 0.26; Sex x Session interaction: *F*_11,110_ = 1.10, *p* = 0.3654; η^2^ = 0.10). Compared with session 1, the rats self-administered more cocaine (Fig. 3B) in sessions 7 (*p* = 0.0051), 8 (*p* = 0.0168), 11 (*p* = 0.0189), and 12 (*p* = 0.0055).

During oral mavoglurant testing, males and females did not differ for cocaine self-administration, and mavoglurant had no effect on cocaine self-administration (Sex effect: *F*_1,10_ = 3.91, *p* = 0.0761; η^2^ = 0.28; Treatment effect: *F*_3,30_ = 0.78, *p* = 0.5143; η^2^ = 0.07; Sex x Treatment interaction: *F*_3,30_ = 1.89, *p* = 0.1530; η^2^ = 0.16; Fig. 3C).

Mavoglurant altered locomotion (Sex effect: *F*_1,10_ = 1.31, *p* = 0.2796; η^2^ = 0.12; Treatment effect: *F*_3,30_ = 3.06, *p* = 0.0431; η^2^ = 0.23; Sex x Treatment interaction: *F*_3,30_ = 0.45, *p* = 0.7184; η^2^ = 0.04; Fig. 3D), regardless of sex. Mavoglurant at 10 mg/kg decreased locomotion compared with 0 mg/kg (*p* = 0.0324).

### LgA - oral

**Fig. 4:**
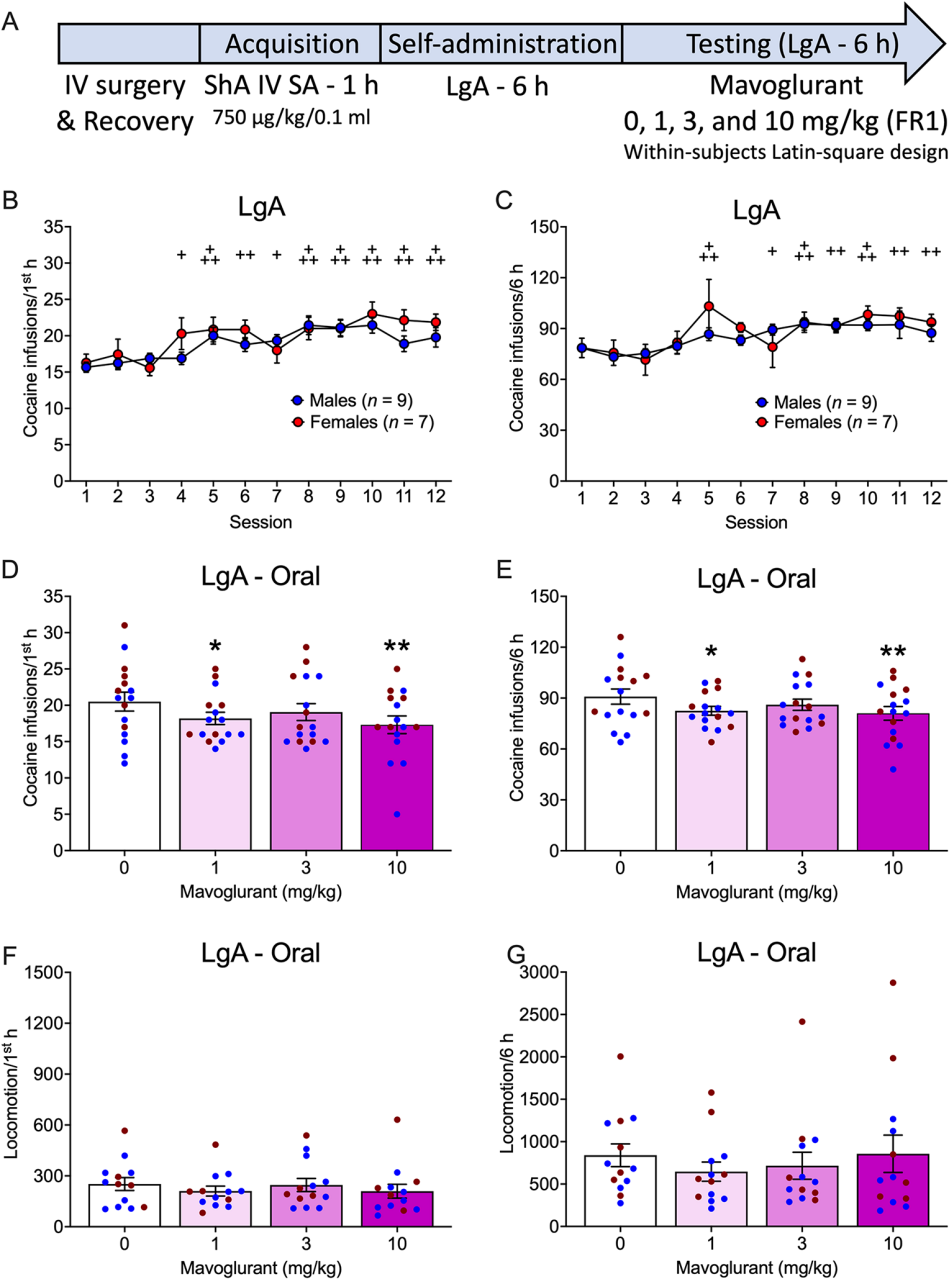
Effect of oral mavoglurant administration on intravenous cocaine self-administration under LgA conditions in rats. **A.** Timeline of the experiment. Acquisition data are not shown. **B, C.** Number of cocaine infusions (750 µg/kg/0.1 ml) before mavoglurant testing in male and female rats in the LgA group in the (**B**) first hour and (**C**) entire 6 h sessions. **D, E.** Number of cocaine infusions in the (**D**) first hour and (**E**) entire 6 h sessions in male and female rats following oral mavoglurant administration. **F, G.** Locomotion in the (**F)** first hour and (**G**) 6 h session in male and female rats following oral mavoglurant administration. The data are expressed as the mean ± SEM. *n* = 9 males (blue symbols), 7 females (red symbols). ^+^*p* < 0.05, ^++^*p* < 0.01, ^+++^*p* < 0.001, difference from session 1, regardless of sex; *p < 0.05, **p < 0.01, difference from 0 mg/kg. IV, intravenous; SA, self-administration; ShA, short-access; FR1, fixed-ratio 1

The timeline of the experiment with oral mavoglurant administration in the LgA group is shown in Fig. 4A. The rats changed their cocaine self-administration during the first hour across sessions, regardless of sex (Sex effect: *F*_1,14_ = 0.75, *p* = 0.4023; η^2^ = 0.05; Session effect: *F*_11,154_ = 8.57, *p* < 0.0001; η^2^ = 0.38; Sex x Session interaction: *F*_11,154_ = 1.24, *p* = 0.2658; η^2^ = 0.08; Fig. 4B). Compared with session 1, rats self-administered more cocaine in sessions 4–12 (*p* < 0.05-0.0001).

Similar results were obtained during the entire 6 h sessions (Sex effect: *F*_1,14_ = 0.68, *p* = 0.4237; η^2^ = 0.05; Session effect: *F*_11,154_ = 7.55, *p* < 0.0001; η^2^ = 0.35; Sex x Session interaction: *F*_11,154_ = 1.08, *p* = 0.3792; η^2^ = 0.07; Fig. 4C). Compared with session 1, rats self-administered more cocaine in sessions 5 (*p* = 0.0005) and 7–12 (*p* < 0.05 − 0.001).

Oral mavoglurant administration decreased cocaine self-administration in the first hour, regardless of sex (Sex effect: *F*_1,14_ = 4.19, *p* = 0.0598; η^2^ = 0.23; Treatment effect: *F*_3,42_ = 3.54, *p* = 0.0226; η^2^ = 0.20; Sex x Treatment interaction: *F*_3,42_ = 0.84, *p* = 0.4790; η^2^ = 0.06). Oral mavoglurant at 3 mg/kg (*p* = 0.0373) and 10 mg/kg (*p* = 0.0057) decreased cocaine self-administration compared with 0 mg/kg in the first hour (Fig. 4D).

Similar results were found for the entire 6 h sessions (Sex effect: *F*_1,14_ = 2.39, *p* = 0.1445; η^2^ = 0.15; Treatment effect: *F*_3,42_ = 3.60, *p* = 0.0210; η^2^ = 0.20; Sex x Treatment interaction: *F*_3,42_ = 2.12, *p* = 0.1116; η^2^ = 0.13). Mavoglurant at 3 mg/kg (*p* = 0.0226) and 10 mg/kg (*p* = 0.0093) reduced cocaine self-administration compared with 0 mg/kg (Fig. 4E).

Mavoglurant did not alter locomotion in rats in the LgA group in the first hour of cocaine self-administration (Sex effect: *F*_1,11_ = 0.72, *p* = 0.4144; η^2^ = 0.06; Treatment effect: *F*_3,33_ = 1.67, *p* = 0.1917; η^2^ = 0.13; Sex x Treatment interaction: *F*_3,33_ = 1.62, *p* = 0.2030; η^2^ = 0.13; Fig. 4F).

Mavoglurant also did not alter locomotion in rats in the LgA group in the entire 6 h sessions (Sex effect: *F*_1,12_ = 0.02, *p* = 0.8818; η^2^ = 0.001; Treatment effect: *F*_3,36_ = 2.24, *p* = 0.1000; η^2^ = 0.16; Sex x Treatment interaction: *F*_3,36_ = 0.42, *p* = 0.7364; η^2^ = 0.03; Fig. 4G).

## Discussion

In the present study, we found that the mGlu5 receptor negative allosteric modulator mavoglurant, administered intraperitoneally or orally, reduced intravenous cocaine self-administration in male and female rats under LgA conditions but not ShA conditions. We did not find sex differences in the effects of mavoglurant.

Rats that were allowed LgA to intravenous cocaine self-administration escalated their cocaine intake across sessions during the first hour and in the entire 6 h sessions. As discussed above, evidence shows that LgA animals exhibit an increase in responding on a progressive-ratio schedule, an increase in responding in the face of punishment, and an increase in reinstatement following extinction than ShA animals (Edwards and Koob 2013). The present results are also consistent with the literature that shows that females self-administer more cocaine than males (Becker and Koob 2016; Towers et al. 2023).

In rats in the LgA group, we found that mavoglurant, administered intraperitoneally or orally, reduced cocaine self-administration. Previous work with mGlu5 receptor blockade is consistent with our observations. Using a FR1 schedule of reinforcement, Kenny et al. (2005) reported that MPEP (3–9 mg/kg) decreased cocaine self-administration in male Wistar rats similarly in the ShA and LgA groups. Martin-Fardon et al. (2009) reported that the negative allosteric modulator MTEP (3 and 10 mg/kg) reduced cocaine self-administration in male rats in 2 h/day sessions. However, MTEP reduced the motivation for cocaine in a progressive-ratio test more in rats in the ShA group than in the LgA group (Hao et al. 2010). Overall, the present results provide evidence that the mGlu5 receptor is also involved in cocaine self-administration under LgA conditions.

We found that mavoglurant, administered intraperitoneally or orally, did not significantly alter cocaine self-administration in rats in the ShA group. However, others have observed a more general decrease in cocaine self-administration with the blockade of mGlu5 receptors. For example, male mice that lacked the mGlu5 receptor gene did not self-administer cocaine, and the mGlu5 receptor negative allosteric modulator MTEP at 10 and 30 mg/kg (intravenous) reduced ShA cocaine self-administration in C57Bl/6J male mice (Chiamulera et al. 2001). In male Wistar rats, MTEP and another mGlu5 receptor negative allosteric modulator, MPEP (1–10 mg/kg), reduced ShA cocaine self-administration (Gould et al. 2016; Hao et al. 2010; Kenny et al. 2005; Tessari et al. 2004). In male Sprague-Dawley rats, two highly selective mGlu5 receptor partial negative allosteric modulators, 2-(2-[3-methoxyphenyl]ethynyl)-5-methylpyridine (M-5MPEP) and 2-(2-[5-bromopyridin-3-yl]ethynyl)-5-methylpyridine (Br-5MPEPy; 10–56 mg/kg), also decreased ShA cocaine self-administration. Mavoglurant is a different chemical compound than the other mGlu5 receptor modulators, which may explain these differences.

Several studies showed a role for the mGlu5 receptor in cocaine reward, behavioral sensitization, and the context-, cue, stress-, and cocaine prime-induced reinstatement of cocaine seeking (for review, see Niedzielska-Andres et al. 2021). The effects of mGlu5 receptor inhibition on the reinstatement of cocaine seeking and extinction learning may involve the ventral and dorsal striatum, endocannabinoids, and some subtypes of protein kinase C and Ca^2+^/calmodulin-dependent protein kinase (Knackstedt et al. 2014; Li et al. 2018; Schmidt et al. 2013; Wang et al. 2013; Zhang et al. 2021).

Previous studies showed that the acute administration of mGlu5 receptor negative allosteric modulators caused a downward shift of the intravenous cocaine self-administration dose-response curve in male rats (Keck et al. 2013, 2014), suggesting that mGlu5 receptor inhibition reduced cocaine’s reinforcer efficacy. Chronic mGlu5 receptor inhibition also caused a downward shift of the cocaine dose-response curve in male squirrel monkeys (Platt et al. 2008) and decreased cue-induced cocaine seeking in male rats that were allowed LgA to cocaine during protracted abstinence (Gobin and Schwendt 2020), providing evidence of a lack of tolerance to mGlu5 receptor inhibition with regard to cocaine-related behaviors. However, the inhibition of mGlu5 receptors elevated brain reward thresholds in male Wistar rats that self-administered cocaine, indicating that MPEP may have induced a negative affective state (Kenny et al. 2005).

The highest dose of oral but not intraperitoneal mavoglurant administration reduced locomotion as measured in the operant chambers during cocaine self-administration in rats in the ShA group. Martin-Fardon et al. (2009) also reported that MTEP (10 mg/kg) caused a transient reduction of locomotion but had no effect on sweetened condensed milk self-administration in male rats, suggesting that the lower locomotor effect of MTEP did not generalize to a palatable reinforcer and that rats can perform operant self-administration despite mild decreases in locomotion. Mavoglurant at any dose or route of administration had no effect on locomotion in the LgA group, suggesting that the effect of mavoglurant in reducing cocaine self-administration in the LgA groups was not attributable to motor effects.

In the present study, we administered mavoglurant intraperitoneally and orally. Drugs that are injected intraperitoneally (similar to orally) undergo first-pass metabolism, but they bypass the stomach and its food content. We tested mavoglurant using oral gavage to achieve face validity, given that mavoglurant is bioavailable when taken orally in humans. The findings obtained herein in male and female rats using two different routes of administration (intraperitoneal and oral) and in two groups with different histories of cocaine self-administration (ShA and LgA) support the hypothesis that mGlu5 receptor is involved in the increased cocaine self-administration associated with extended access.

In the present study, we found similar effects of mavoglurant in decreasing LgA cocaine self-administration in male rats and intact (non-ovariectomized) female rats. We found only two studies of the mGlu5 receptor that included female rodents. Ovariectomized female Sprague-Dawley rats that received estradiol exhibited an enhancement of the escalation of cocaine self-administration in LgA sessions compared with ovariectomized females that did not receive estradiol supplementation. MPEP administration before estrogen supplementation in a 2 day on/2 day off schedule during the escalation phase blocked the estradiol-induced enhancement of cocaine intake (Martinez et al. 2016). MPEP did not affect escalation in ovariectomized female Sprague-Dawley rats that did not receive estradiol, and intact (non-ovariectomized) females were not tested (Martinez et al. 2016). Female Sprague-Dawley rats in estrus exhibited an increase in the cue-induced reinstatement of cocaine seeking compared with females in other estrous stages and males. An injection of MTEP in the basolateral amygdala reduced cue-induced cocaine seeking only in females that were in estrus and not in other estrous stages (Corbett et al. 2023). Thus, sex hormones may modulate the effect of mGlu5 receptor inhibition on cocaine-related behaviors.

Mavoglurant has been tested in several clinical studies for different conditions. Its safety and tolerability have been established. A search of ClinicalTrials.gov (January 24, 2024) indicated that a few studies have been completed or are ongoing for alcohol and cocaine use. A randomized, double-blind, placebo-controlled study (NCT03242928) in people with CUD showed that mavoglurant was superior to placebo with regard to the proportion of cocaine use days and positive urine measurements of benzoylecgonine, the main metabolite of cocaine. No serious adverse events were observed in the mavoglurant group, and a similar incidence of other non-serious adverse events was found between the mavoglurant and placebo groups.

In conclusion, the present findings that mGlu5 receptor inhibition reduced extended-access cocaine self-administration support the ongoing clinical development of mavoglurant to evaluate its potential therapeutic benefits for CUD.
